# Virginia Medical and Surgical Journal

**Published:** 1853-01

**Authors:** 


*Virginia Medical and Surgical Journal.*—We have received a prospectus of a Medical Journal, to be published in Richmond, Virginia, under the above title. The Editors are Drs. George A. Otis and H. L. Thomas. Each number will consist of eighty pages, 8vo., containing original papers, memoirs, reports of cases, an account of the transactions of medical societies in Virginia; a summary of improvements and discoveries in the medical science, reviews, bibliographical notices, &c. The work will be published monthly, at $5 00 per annum. We wish the editors success in their undertaking.

